# Supplementary material for: From sequence to enzyme mechanism using multi-label machine learning
Source: BMC Bioinformatics. 2014 May 19;15:150. doi: 10.1186/1471-2105-15-150 (PMC4229970; doi:10.1186/1471-2105-15-150)
Supplement: Additional file 2 — Java code of ml2db. Additional file ml2db_code.tar.gz contains the Java source code to run the multi-label machine learning experiments and save the results to database. The code’s Javadoc is included. [file 1471-2105-15-150-S2.zip › additional file 2/ml2db/utils/doc/cern/colt/function/class-use/DoubleFunction.html]

Uses of Interface cern.colt.function.DoubleFunction


JavaScript is disabled on your browser.


- Overview
- Package
- Class
- Use
- Tree
- Deprecated
- Index
- Help

- Prev
- Next

- Frames
- No Frames

- All Classes

## Uses of Interface cern.colt.function.DoubleFunction

- Packages that use DoubleFunction

  | Package | Description |
  | --- | --- |
  |  |  |
  | --- | --- |
  | cern.jet.random |  |
  | cern.jet.random.engine |  |
  | uk.ac.standrews.utils.main.stats |  |
- - ### Uses of DoubleFunction in cern.jet.random

    Classes in cern.jet.random that implement DoubleFunction

    | Modifier and Type | Class and Description |
    | --- | --- |
    |  |  |
    | --- | --- |
    | `class` | `AbstractContinousDistribution` Abstract base class for all continous distributions. |
    | `class` | `AbstractDistribution` Abstract base class for all random distributions. |
    | `class` | `Pareto` Pareto Distribution. |
  - ### Uses of DoubleFunction in cern.jet.random.engine

    Classes in cern.jet.random.engine that implement DoubleFunction

    | Modifier and Type | Class and Description |
    | --- | --- |
    |  |  |
    | --- | --- |
    | `class` | `MersenneTwister` MersenneTwister (MT19937) is one of the strongest uniform pseudo-random number generators known so far; at the same time it is quick. |
    | `class` | `RandomEngine` Abstract base class for uniform pseudo-random number generating engines. |
  - ### Uses of DoubleFunction in uk.ac.standrews.utils.main.stats

    Classes in uk.ac.standrews.utils.main.stats that implement DoubleFunction

    | Modifier and Type | Class and Description |
    | --- | --- |
    |  |  |
    | --- | --- |
    | `class` | `PseudoTruncatedPareto` A truncated pareto distribution which stores a big lists of power-law extracted random numbers. |
    | `class` | `TruncatedPareto` A utility class to extract from a truncated Pareto (power-law, long-tail) distribution. |

- Overview
- Package
- Class
- Use
- Tree
- Deprecated
- Index
- Help

- Prev
- Next

- Frames
- No Frames

- All Classes
